# Multistage feature fusion knowledge distillation

**DOI:** 10.1038/s41598-024-64041-4

**Published:** 2024-06-11

**Authors:** Gang Li, Kun Wang, Pengfei Lv, Pan He, Zheng Zhou, Chuanyun Xu

**Affiliations:** 1 School of Artificial Intelligence, Chongqing University of Technology, Chongqing, 401135 China; 2 College of Computer and Information Science, Chongqing Normal University, Chongqing, 401331 China

**Keywords:** Knowledge distillation, Label classification, Multistage, Feature fusion, Attention mechanism, Computer science, Computational science

## Abstract

Generally, the recognition performance of lightweight models is often lower than that of large models. Knowledge distillation, by teaching a student model using a teacher model, can further enhance the recognition accuracy of lightweight models. In this paper, we approach knowledge distillation from the perspective of intermediate feature-level knowledge distillation. We combine a cross-stage feature fusion symmetric framework, an attention mechanism to enhance the fused features, and a contrastive loss function for teacher and student models at the same stage to comprehensively implement a multistage feature fusion knowledge distillation method. This approach addresses the problem of significant differences in the intermediate feature distributions between teacher and student models, making it difficult to effectively learn implicit knowledge and thus improving the recognition accuracy of the student model. Compared to existing knowledge distillation methods, our method performs at a superior level. On the CIFAR100 dataset, it boosts the recognition accuracy of ResNet20 from 69.06% to 71.34%, and on the TinyImagenet dataset, it increases the recognition accuracy of ResNet18 from 66.54% to 68.03%, demonstrating the effectiveness and generalizability of our approach. Furthermore, there is room for further optimization of the overall distillation structure and feature extraction methods in this approach, which requires further research and exploration.

## Introduction

In recent years, convolutional neural networks(CNNs) in the field of deep learning have greatly advanced the field of computer vision and have found extensive applications in tasks such as image classification^1–3^, object detection^4–6^, and semantic segmentation^7–9^. However, due to the computational constraints and memory limitations of edge computing devices, deploying large convolutional network models poses challenges. Balancing computational costs with model performance remains a highly challenging problem, and knowledge distillation^10–13^ provides an effective solution. Knowledge distillation, in the form of a teacher supervising a student, transfers knowledge from a large model to a smaller model, significantly improving the student model’s performance without increasing the computational complexity. This approach is simple and effective and has been widely applied to convolutional networks and classification tasks.

Common knowledge distillation methods are generally divided into two categories. The first category is based on soft-label classification knowledge. These methods utilize different temperature settings to soften the classification labels of the teacher and student networks. By reducing the final knowledge discrepancy in the softened labels, they aim to enhance the recognition accuracy of the student network. The second category is based on intermediate layer features. Typically, the teacher network and student network share some structural and learning process similarities. The student network can learn the implicit knowledge present in the teacher network’s intermediate feature layers, resulting in an improved learning process and, consequently, an enhancement in its own accuracy. This approach achieves improved accuracy for smaller models by transferring knowledge through multiple stages of intermediate feature layers.

**Figure 1:**
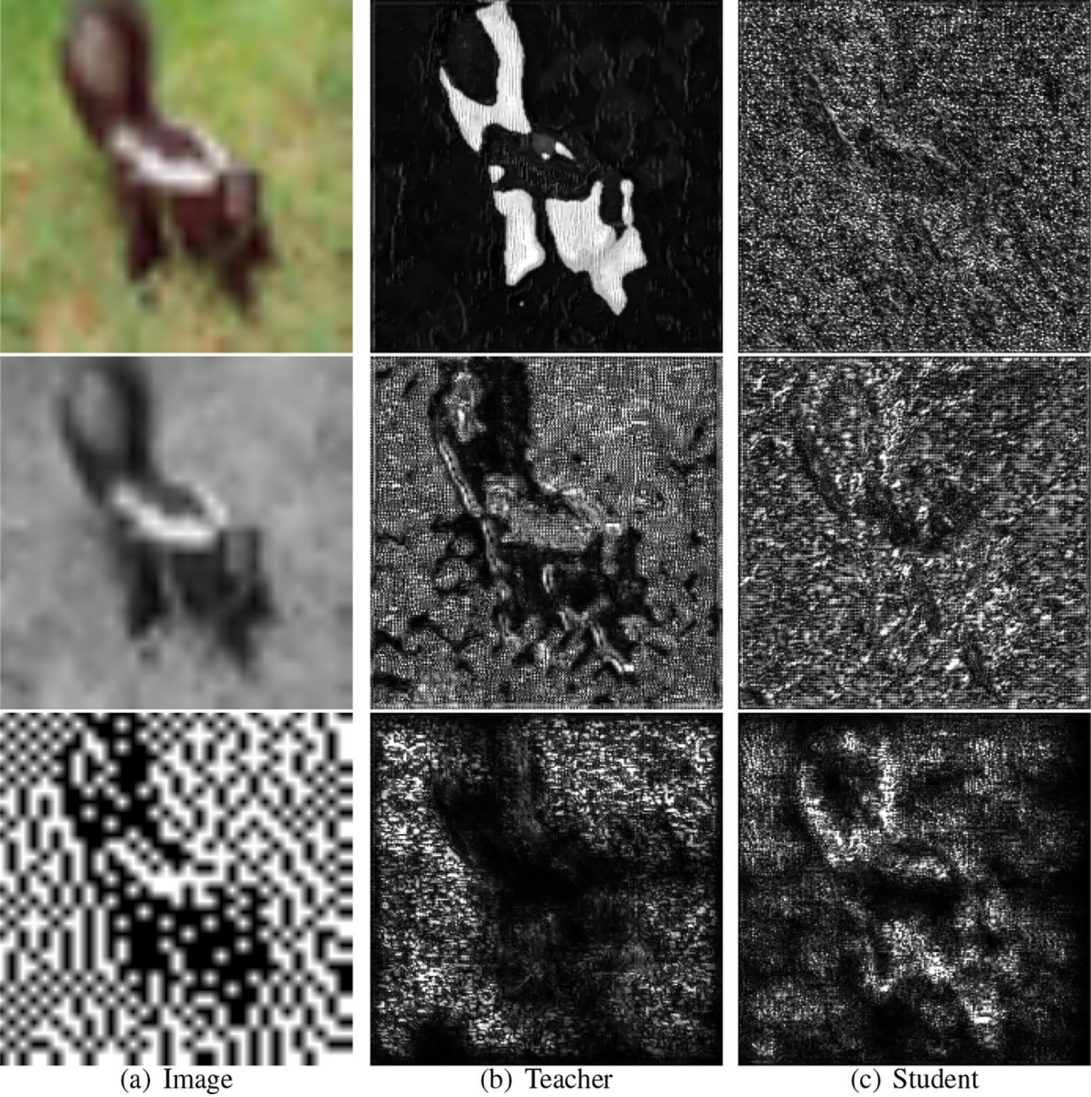
Model feature gradient maps at different stages^14^. (**a**) Displays the original image, grayscale image, and binary image. (**b**,**c**) Shows the multistage feature maps of ResNet56 and ResNet20, respectively.

**Figure 2:**
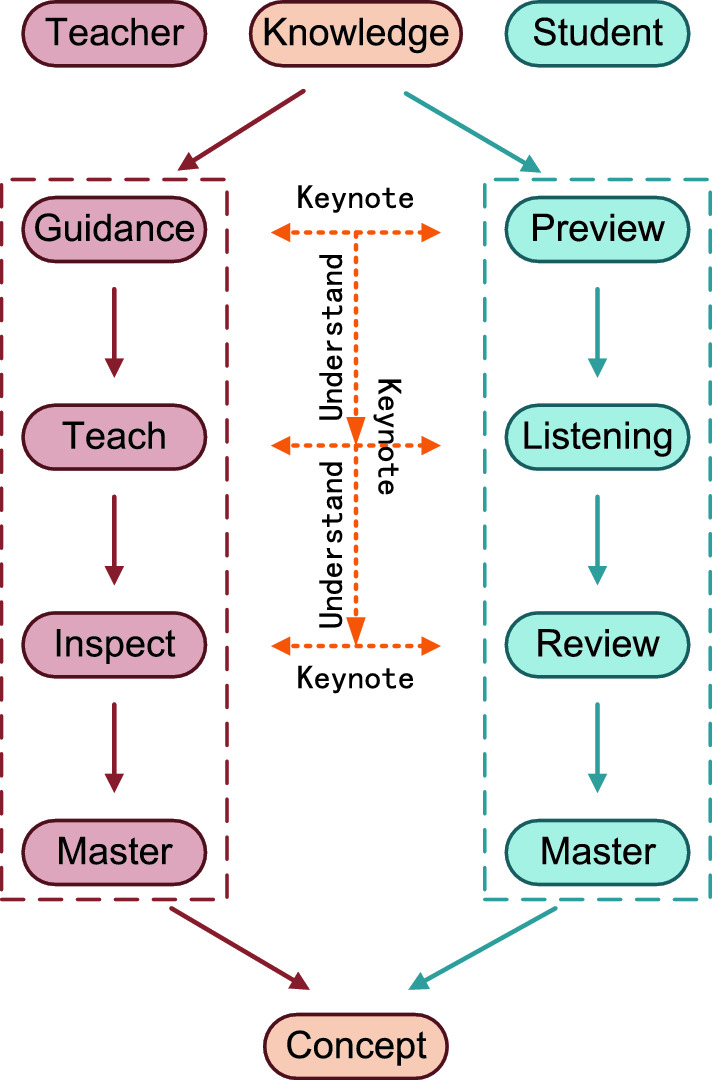
The overall idea of this experiment.

In addition, knowledge distillation is a type of precision improvement method for general models, which has various implementation methods and high competition. The feature distributions between teacher and student networks at the same stage often exhibit significant differences, and different stages of the same network also focus on distinct aspects. As shown in Fig. 1, deep features emphasize conceptual information, while shallow features emphasize textural information. Therefore, extracting useful knowledge from the irregular intermediate feature distribution of teacher and student networks is a challenging research problem. In order to effectively address this issue, Fig. 2 shows the source of our method ideas. There are four teaching phenomena in the actual teaching process. 1. Teachers teach students in stages. 2. The methods taught by teachers and the methods learned by students are consistent. 3. Early guidance from teachers can guide students in their later learning. 4. Knowledge transmission and learning have core and universally applicable parts. The four concepts based on real-life teaching phenomena mentioned above are the origin of our design ideas for knowledge distillation methods.

**Figure 3:**
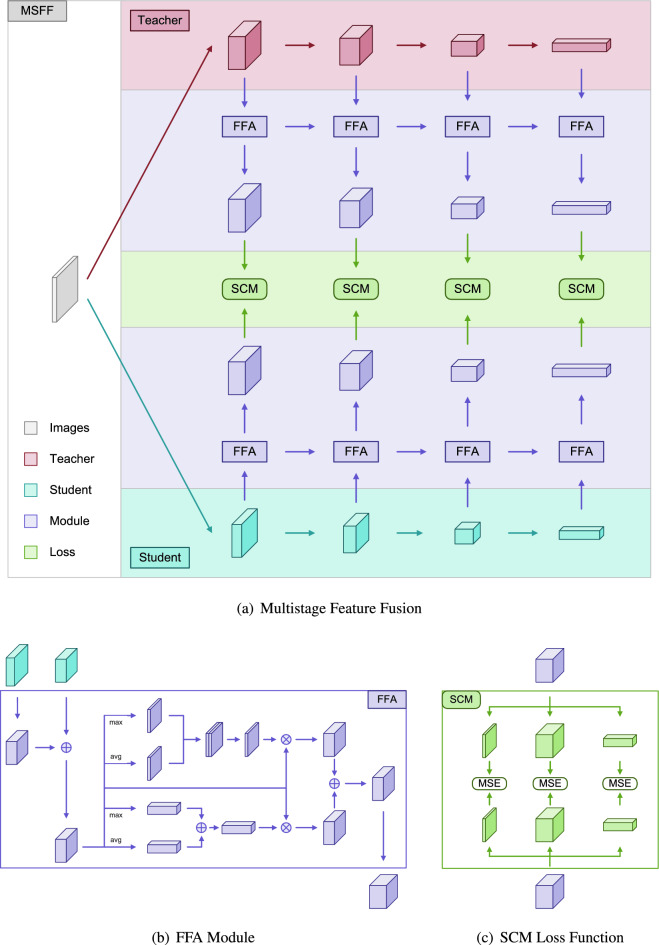
The complete structure of the multistage feature fusion knowledge distillation method is illustrated, consisting of three components: (**a**) shows the multistage feature fusion framework; (**b**) demonstrates the construction of the FFA Module; (**c**) illustrates the structure of the SCM loss function.

Therefore, based on the four teaching phenomena mentioned above, we take the teaching approach of teachers imparting knowledge to students in reality as the starting point, and design multistage feature fusion distillation frameworks corresponding to different stages of teaching modes, which are used to achieve the fusion attention mechanism of knowledge cross stage flow and the spatial and channel loss function to verify actual learning effects. Through these three innovative points, we have applied specific teaching concepts in the field of feature layer knowledge distillation, achieving a universal and reliable feature information fusion and teaching method. Figure 3 shows the overall distillation framework of our method. We designed a multi-stage feature fusion framework, a cross stage feature fusion attention mechanism, and spatial and channel loss functions. This achieves the beneficial distillation of the global and local effects of the teacher network on the student network. The inspiration provided by our method lies in combining knowledge distillation with real-life teaching methods, which has practical significance in further guiding and improving real-life teaching methods. The multi-stage feature fusion framework achieves knowledge transfer between teacher models and student models from shallow texture features to deep conceptual features, thereby achieving the effect of early knowledge guidance for middle and later learning, as well as layer by layer knowledge guidance. The cross stage feature fusion attention mechanism builds a bridge for knowledge to flow from shallow to deep layers by integrating feature knowledge from adjacent stages, and extracts general and prominent features of teacher and student networks from parallel channel attention and spatial attention methods, realizing the teacher’s teaching of core and general knowledge to students. In order to more intuitively and effectively compare the differences between teachers and students regarding the extracted features, based on the attention module of feature extraction, we achieved effective comparison between features from three aspects: direct comparison, spatial comparison, and channel comparison, achieving better results. In summary, we have provided some feasible research ideas and academic references for other researchers.

The above content introduces challenges related to the disparity in feature knowledge distribution between the teacher and student, making it difficult for the student network to directly learn the teacher network’s feature implicit knowledge. To address the above issues, we design a multistage feature fusion knowledge distillation method, as illustrated in Fig. 3. This approach leverages a multistage feature fusion framework, cross-stage feature fusion attention modules, and spatial and channel contrastive loss functions for features fused at the same stage. These components enable the student network to learn hidden knowledge from multiple stages of the teacher network, resulting in a highly valuable enhancement of model accuracy. The main contributions of this paper are as follows:

1. We introduce a multistage feature fusion framework (MSFF) that facilitates the transfer of knowledge from shallow to deep layers across stages. MSFF adopts a multi-level symmetric framework structure, allowing teachers and students to impart and learn knowledge in the same way, ensuring that the multi-level knowledge of the teacher model can be effectively transmitted to the student model.

2. We propose the feature fusion attention module (FFA) based on spatial and channel attention. This module aims to extract the average and prominent knowledge of spatial and channel features in parallel, and fuse the features at the end to achieve the condensation of feature knowledge.

3. We introduce the spatial and channel mean squared error loss (SCM) as a counterpart to the FFA. SCM can compare the feature differences between teachers and students in terms of original features, spatial features, and channel features.

## Related work

### Knowledge distillation

Knowledge distillation (KD), as initially proposed by Hinton et al.^10^, aims to supervise the training convergence of a student network, a smaller model, with a teacher network, a larger model. This method controls the extent of knowledge transfer between two networks using a temperature parameter, T, to control the transfer of soft-label dark knowledge. This approach has given rise to variations, including intermediate feature layer distillation and multistage soft label distillation.

In the context of intermediate feature layer distillation, the challenge of inconsistent multistage feature knowledge distribution is a critical issue. FitNet^11^ employs squared distance constraints to measure the similarity of intermediate layer features between teacher and student networks. AT^15^ uses multi-layer attention maps to extract features between the teacher network and the student network, and builds a knowledge transfer mechanism between the two. CC^16^ proposed a correlation congruence method to reduce the correlation consistency distribution between teachers and students across multiple sample instances, and improve the distribution consistency between student models and teacher models in the classification output of multiple instances. AB^17^ proposed a method for knowledge transfer by extracting the activation boundaries formed by hidden neurons, which enables students to learn the separation boundaries between different activation regions formed by each neuron in the teacher, thereby reducing the differences between the student network and the teacher network. FT^18^ proposed two convolutional modules, the reader and the translator, which are used to extract the feature information of teachers and the translator to extract the feature information of students. Through distillation training, the differences between the two modules are reduced, achieving the imitation and learning of the teacher network by the student network. NST^19^ proposed a new KT loss function to minimize the maximum average difference in neuron feature distribution between the teacher model and the student model, significantly improving the performance of the student model. CRD^20^ is a knowledge distillation method based on contrastive learning, which preserves mutual information between teachers and students by optimizing the distillation loss function. OFD^21^ uses a novel distance function and edge residual function to distill essential information between teacher and student networks. ReviewKD^22^ utilizes a multilevel composite knowledge approach to transfer dark knowledge at the feature level, achieving state-of-the-art performance. In the realm of multistage knowledge distillation methods, TSKD^23^ effectively enhances the testing accuracy of student networks through multistage guidance from teacher networks. OtO^24^ employs a joint multistage to multistage training approach between teacher and student networks, achieving significant improvements in multistage knowledge distillation.

In contrast to the above methods, our experiment utilizes a multistage feature fusion knowledge distillation approach. This approach effectively addresses the challenges of feature distribution disparities in knowledge transfer, resulting in a substantial enhancement of recognition accuracy for lightweight models.

### Attention mechanism

The essence of attention mechanisms lies in extracting key information from features through element-to-element similarity.

In the field of computer vision, two primary types of attention mechanisms are spatial attention and channel attention. Among these, SENet^25^ uses global average pooling to compress channel information, thereby enhancing feature representation in the channel dimension. SRM^26^ employs an adaptive style calibration module based on global average pooling and global standard deviation pooling to capture global feature information. It is a lightweight structure with a small number of parameters. GENet^27^ combines interpolation methods to capture correlations between feature maps at different spatial positions, enabling the capture of global contextual information. RGA^28^ utilizes symmetric relationships between different features to capture global correlations and semantic information, and is applicable in both spatial and channel dimensions. CBAM^29^ combines channel attention and spatial attention mechanisms to extract global maximum and average feature information.

The method used in this experiment, FFA, employs a parallel structure by extracting the maximum and average information of global features separately in the spatial and channel dimensions to enhance features. It is a simple and effective structure.

## Method and principles

In this paper, we design a multistage feature fusion knowledge distillation method that focuses on a symmetric framework for cross-stage feature fusion, an attention mechanism to enhance the fused features, and a spatial and channel-based contrastive loss function for teacher and student networks at the same stage. This method achieves fused multistage feature knowledge transfer from the teacher network to the student network, as illustrated in Fig. 1.The overall methodology can be found in Algorithm 1.

**Algorithm 1:**
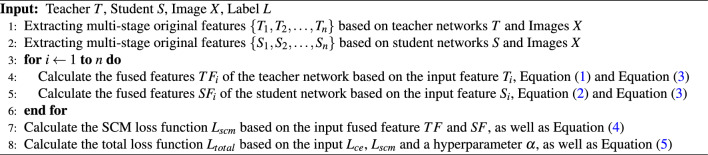
Multistage feature fusion knowledge distillation

### Multistage feature fusion framework

The multistage feature fusion framework used in this paper is a symmetric network architecture that facilitates symmetric teaching and learning between the teacher network and the student network, enabling effective knowledge transfer at the intermediate feature layers.

Both the teacher network *T* and the student network *S* consist of *n* feature output stages and *n* corresponding feature fusion attention modules $FFA_{i}$, where $i\in n$. The *i*-th layer features are denoted as $T_i$ and $S_i$. In this framework, the first feature fusion module has only one input port, while the subsequent stages have two input ports. The output features of the *i*-th fusion layer are denoted as $F_{i}^1$ and $F_{i}^2$. The final output stage has only one output port, whereas the earlier stages have two output ports. The size and channel count of the fused output features $F_{i}^1$ in a single stage match the unfused features of the corresponding stage in the teacher network.

Based on this framework, the feature fusion formula for the teacher network can be expressed as follows:

1 $$\begin{aligned} (Ft_{i}^1,Ft_{i}^2)={\left\{ \begin{array}{ll}FFA_{i}(T_{i}),\quad i=0 \\ FFA_{i}(T_{i-1}^1, T_{i}),\quad i<n \end{array}\right. } \end{aligned}$$

The feature fusion formula for the student network can be expressed as follows:

2 $$\begin{aligned} (Fs_{i}^1,Fs_{i}^2)={\left\{ \begin{array}{ll}FFA_{i}(S_{i}),\quad i=0 \\ FFA_{i}(S_{i-1}^1, S_{i}),\quad i<n \end{array}\right. } \end{aligned}$$

### Feature fusion attention module

In the feature fusion attention module *FFA*, the dimensions and channel counts of two different stage features $I_{1}$ and $I_{2}$ are generally not the same. Here, a convolution and normalization module *M* is used to adjust the size and channel count of the input feature $I_{1}$ to match that of $I_{2}$. This adjusted feature *I* is obtained via addition. Subsequently, with the parallel channel attention mechanism *Ac* and spatial attention mechanism *As*, the parallel results are added to obtain the fused feature *F*. After convolution and normalization, two output features $F^1$ and $F^2$ are generated, which generally have different dimensions and channel counts. The feature fusion module $FFA_{1}$ takes only $I_{2}$ as input, while the feature fusion module $FFA_{n}$ has only $F_{1}$ as output.

The formula for *FFA* is as follows:

3 $$\begin{aligned} (F^1,F^2)=As(M(I_{1})+I_{2})+Ac(M(I_{1})+I_{2}) \end{aligned}$$

### Contrastive loss function

During the training phase of the student network, the spatial and channel mean squared error loss function $L_{scm}$ is used for comparisons between the fused features of the same stage. This loss function divides the corresponding $TF_{i}$ and $SF_{i}$ of the teacher network and student network for the *i*-th stage fused feature $F^2_{i}$ into three parts for $L_{mse}$ similarity matching:

1. No processing.

2. Channel compression without altering the feature spatial size, resulting in $TF^1_{i}$ and $SF^1_{i}$.

3. Spatial compression without altering the channel count, resulting in $TF^2_{i}$ and $SF^2_{i}$.

This, combined with the weight adjustment hyperparameter $\lambda$, constitutes *n* stage fused feature comparison functions. The formula is as follows:

4 $$\begin{aligned} \begin{aligned} L_{scm}&= L_{mse}(TF_{i},SF_{i}) + \lambda L_{mse}(TF_{i}^1,SF_{i}^1) + \lambda L_{mse}(TF_{i}^2,SF_{i}^2) \end{aligned} \end{aligned}$$

In addition, it is combined with the cross-entropy loss function $L_{ce}$ between the true labels and the student’s classification results, along with the weight adjustment hyperparameter $\alpha$. This constitutes the complete loss function, and the formula is as follows:

5 $$\begin{aligned} L_{total}=L_{ce}+\alpha L_{scm} \end{aligned}$$

## Experiments and results

### Experimental parameter details

The CIFAR-100 classification dataset^35^ consists of 100 categories with images of size 32$\times$32. The dataset includes 50,000 training images and 10,000 validation images. The experiments were conducted using various representative network architectures, including ResNet v2^36^, VGG^37^, ResNet^38^, WideResNet^39^, MobileNet^40^, and ShuffleNet^41,42^. The training strategies followed the definitions in^43^, with a batch size of 64 and SGD. The weight decay and momentum were set to 5e-4 and 0.9, respectively. The learning rate was defined as 0.01 for ShuffleNet and MobileNetV2, while it was defined as 0.05 for the other models. The training process ran for 240 epochs, and the learning rate was divided by 10 at the 150th, 180th, and 210th epochs.

**Table 1:** Experimental results on the CIFAR-100 dataset with the teacher and student having the same network architecture.

Teacher	resnet56	resnet110	resnet32x4	WRN-40-2	WRN-40-2	VGG13
ACC	72.34	74.31	79.42	75.61	75.61	74.64
Student	resnet20	resnet32	resnet8x4	WRN-40-1	WRN-16-2	VGG8
ACC	69.06	71.14	72.50	71.98	73.26	70.36
KD^10^	70.66	73.08	73.33	73.54	74.92	72.98
FitNet^11^	69.21	71.06	73.50	72.24	73.58	71.02
AT^15^	70.55	72.31	73.44	72.77	74.08	71.43
VID^30^	70.38	72.61	73.09	73.30	74.11	71.23
SP^31^	69.67	72.69	72.94	72.43	73.83	72.68
CC^16^	69.63	71.48	72.97	72.21	73.56	70.71
AB^17^	69.47	70.98	73.17	72.38	72.50	70.94
FT^18^	69.84	72.37	72.86	71.59	73.25	70.58
NST^19^	69.60	71.96	73.30	72.24	73.68	71.53
PKT^32^	70.34	72.61	73.64	73.45	74.54	72.88
RKD^33^	69.61	71.82	71.90	72.22	73.35	71.48
CRD^20^	71.16	73.48	75.51	74.14	75.48	73.94
OFD^21^	70.98	73.23	74.95	74.33	75.24	73.95
ReviewKD^22^	71.89	73.89	75.63	75.09	76.12	74.84
DKD^34^	71.97	74.11	76.32	74.81	76.24	74.68
**MSFF**	71.34	73.24	74.67	74.43	75.60	73.92
$\uparrow$	2.28	2.10	2.17	2.45	2.34	3.56

**Table 2:** Experimental results on the CIFAR-100 dataset with the teacher and student having different network architectures.

Teacher	resnet32$\times$4	resnet32$\times$4	WRN-40-2	VGG13
ACC	79.42	79.42	75.61	74.64
Student	ShuffleNet-V1	ShuffleNet-V2	ShuffleNet-V1	MobileNet-V2
ACC	70.50	71.82	70.50	64.60
KD^10^	74.07	74.45	74.83	67.37
FitNet^11^	73.59	73.54	73.73	64.14
AT^15^	71.73	72.73	73.32	59.40
VID^30^	73.38	73.40	73.61	65.56
SP^31^	73.48	74.56	74.52	66.30
CC^16^	71.14	71.29	71.38	64.86
AB^17^	73.55	74.31	73.34	66.06
FT^18^	71.75	72.50	72.03	61.78
NST^19^	74.12	74.68	74.89	58.16
PKT^32^	74.10	74.69	73.89	67.13
RKD^33^	72.28	73.21	72.21	64.52
CRD^20^	75.11	75.65	76.05	69.73
OFD^21^	75.98	76.82	75.85	69.48
ReviewKD^22^	77.45	77.78	77.14	70.37
DKD^34^	76.45	77.07	76.70	69.71
**MSFF**	75.54	76.09	76.23	67.56
$\uparrow$	5.04	4.27	5.73	2.94

The TinyImageNet classification dataset^44^ contains 200 categories, and the images have a size of 64x64. The dataset comprises 10,000 training images and 10,000 validation images. For this dataset, the ResNet v2^36^ model was used, and the same training strategy as CIFAR-100 was applied. The experimental framework used in this paper was modified based on the framework used in^34^.

**Table 3:** Transfer experiment results with the teacher and student combination of WRN-40-2 and WRN-40-1.

Datasets	Baseline	KD^10^	VID^30^	PKT^32^	RKD^33^	CRD^20^	MSFF
CIFAR100 $\rightarrow$ STL-10	63.60	63.68	65.04	63.48	64.39	**66.70**	66.14
CIFAR100 $\rightarrow$ TinyImageNet	27.06	27.70	26.87	27.70	28.01	**30.24**	29.06

**Table 4:** Experimental results on the TinyImageNet dataset with a teacher network of ResNet34.

Student	Baseline	KD^10^	FitNet^11^	AT^15^	SP^31^	VID^30^	CRD^20^	AFD^45^	MSFF
ResNet18	64.40	66.54	67.18	66.66	67.56	67.56	67.66	**68.10**	68.03
ResNet34	66.40	68.80	66.18	67.16	68.36	68.08	68.96	**69.58**	68.98

**Table 5:** The experimental results of various frameworks under different stage combinations.

Teacher / Student	resnet56 / resnet20	VGG13 / VGG8
Only First Stage	69.41	71.23
Only Last Stage	71.12	72.98
First and Last Stages	71.14	73.45
All Stages	71.34	73.92

**Table 6:** Results of framework and module ablation experiments.

Framework and Modules				resnet56
MS	MSF	SCM	FFA	resnet20
$\checkmark$				70.51
$\checkmark$	$\checkmark$			70.87
$\checkmark$	$\checkmark$	$\checkmark$		71.01
$\checkmark$	$\checkmark$		$\checkmark$	71.24
$\checkmark$	$\checkmark$	$\checkmark$	$\checkmark$	71.34

### Comparative experiments

#### Results on the CIFAR-100 dataset

On the CIFAR-100 dataset, we conducted multiple experiments to evaluate the effectiveness and generalizability of the MSFF method. As shown in Tables 1 and 2, compared to other distillation methods, the MSFF demonstrates broad applicability and achieves competitive accuracy improvements on various lightweight network models. This allows the student network to learn valuable knowledge from the teacher network. Compared with the commonly used distillation methods CRD and OFD in recent years, our method is at the same level. Compared with the latest achievements in the same field, ReviewKD and DKD, our method achieves slightly less improvement, but it is competitive.

In the inference phase, by pruning the teacher network and the multistage feature fusion framework while retaining only the architecture of the student network, we were able to improve the accuracy of ResNet20 from $69.06\%$ to $71.34\%$, an increase of 2.28 percentage points, and the accuracy of VGG8 from $70.36\%$ to $73.92\%$, an increase of 3.56 percentage points. Table 1 demonstrates the effectiveness and generalizability of the MSFF method when the teacher and student models are of the same type. It achieves competitive performance on the majority of the compared models, increasing the accuracy of ResNet32 from $71.14\%$ to $73.24\%$, an increase of 2.1 percentage point, and the accuracy of WRN-40-1 ranged from $71.98\%$ to $74.43\%$, an increase of 2.45 percentage points. Table 2 illustrates the effectiveness and generalizability of the MSFF method when the teacher and student models are of different types, achieving significant accuracy improvements. This approach increases the accuracy of ShuffleNet-V1 from $70.50\%$ to $76.23\%$, an increase of 5.73 percentage points, and the accuracy of MobileNet-V2 from $64.60\%$ to $67.56\%$, an increase of 2.96 percentage points.

The experiments shown in Table 3 were conducted on the CIFAR-100 dataset with the teacher network WRN-40-2. Various knowledge distillation methods were used to train student network WRN-40-1, and the resulting WRN-40-1 models were subsequently transferred to the STL-10 and TinyImageNet datasets to assess their accuracy. The data in the table show that the knowledge distillation method proposed in this paper achieved valuable accuracy improvements compared to the baseline accuracy and other knowledge distillation methods. This further confirms the effectiveness and generalizability of the proposed method.

### Results on the TinyImageNet dataset

With respect to images in the TinyImageNet dataset, we further examined the effectiveness and generalizability of the MSFF method. Table 4 presents the comparative results of the MSFF and various distillation methods on ResNet34 and ResNet18. The experiments show that our method is highly effective, with accuracy improvements exceeding those of most other methods. It can increase the accuracy of ResNet18 from $64.40\%$ to $68.03\%$, an improvement of 3.63 percentage points. Furthermore, the approach of teaching using the same model is also highly effective, increasing the accuracy of ResNet34 from $66.40\%$ to $68.98\%$, an improvement of 2.58 percentage points.

### Multistage architecture and module ablation experiments

In order to better validate the effectiveness of the method proposed in this paper, we conducted multi-stage comparative experiments and module ablation experiments in this chapter. The multi-stage comparative experiments focused on verifying the differences in feature learning ability under different stage feature combinations, while the module ablation experiments verified the differences in the contribution of the multi-stage feature fusion framework, cross stage feature fusion attention mechanism, and loss function to the overall improvement effect of the multi-stage feature fusion knowledge distillation proposed in this paper. The specific experimental process is as follows:

Table 5 shows four different combinations of stages: the first stage, the last stage, both the first and last stages, and all stages. The results of distillation experiments based on two different teacher models and student models indicate that different stages have varying efficiencies of knowledge transfer. The complete MSFF method achieves balanced optimization across multiple stages and effectively addresses the issue of inconsistent feature distributions in different stages, resulting in the best model accuracy in the experiments.

To further demonstrate the effectiveness of the MSFF method, various ablation experiments were conducted. Table 6 presents a comparison of framework and module ablation experiments for the multi-stage feature fusion knowledge distillation method to determine the impact of different modules on the experimental results. In the table, MS represents multistage direct comparison without using the fusion framework, it can be seen that the recognition result of the student network is 70.51%, which is the lowest value in the list. MSF indicates multi-stage direct comparison using the fusion framework, the recognition accuracy of the corresponding student model obtained using MS and MSF methods is 70.87%, slightly better than the MS method, proving the effectiveness of multi-stage feature extraction. SCM represents the use of the spatial and channel contrastive loss function, by using MS, MSF, and SCM simultaneously, the recognition accuracy of the student network has further increased, reaching 71.01%, proving the effectiveness of the SCM loss function. FFA stands for using the feature fusion attention module, by overlaying FFA on the basis of MS and MSF, the recognition performance of the student model reached 71.24%, which has significantly improved on the basis of MS and MSF, proving that using feature fusion methods to extract useful feature knowledge is very beneficial. After using all frameworks and modules, the accuracy of student model recognition reached 71.34%, achieving the best student model recognition results. Ablation experiments confirm the effectiveness of both the framework and module combinations, as they can effectively improve the student model’s recognition performance compared to the baseline model and address the issue of inconsistent feature distributions across multiple stages.

## Conclusion

In this paper, we introduce the concept of multistage feature fusion knowledge distillation, which addresses the issue of feature distribution mismatch between teacher and student networks across multiple stages. Starting from a symmetric framework for cross-stage feature fusion, enhancing the fused features through attention mechanisms, and employing spatial and channel contrastive loss functions at the same stage between the teacher and student networks, we successfully achieved effective global knowledge transfer from the teacher network to the student network. The experimental results demonstrated that the MSFF method exhibits impressive versatility and achieves notable performance improvements. However, compared to existing methods, there remains room for further enhancements in terms of framework structure and feature extraction.

## Data Availability

The data that support the findings of this study are available on request from the corresponding author, Chuanyun Xu, upon reasonable request.
